# RSI-CB: A Large-Scale Remote Sensing Image Classification Benchmark Using Crowdsourced Data

**DOI:** 10.3390/s20061594

**Published:** 2020-03-12

**Authors:** Haifeng Li, Xin Dou, Chao Tao, Zhixiang Wu, Jie Chen, Jian Peng, Min Deng, Ling Zhao

**Affiliations:** School of Geosciences and Info-Physics, Central South University, Changsha 410083, China; lihaifeng@csu.edu.cn (H.L.); 175011004@csu.edu.cn (X.D.); kingtaochao@csu.edu.cn (C.T.); zhixiangwu17@gmail.com (Z.W.); bluesleon@gmail.com (J.C.); PengJ2017@csu.edu.cn (J.P.); dengmin@csu.edu.cn (M.D.)

**Keywords:** remote sensing image classification, benchmark, crowdsourced data, deep convolution neural network

## Abstract

Image classification is a fundamental task in remote sensing image processing. In recent years, deep convolutional neural networks (DCNNs) have experienced significant breakthroughs in natural image recognition. The remote sensing field, however, is still lacking a large-scale benchmark similar to ImageNet. In this paper, we propose a remote sensing image classification benchmark (RSI-CB) based on massive, scalable, and diverse crowdsourced data. Using crowdsourced data, such as Open Street Map (OSM) data, ground objects in remote sensing images can be annotated effectively using points of interest, vector data from OSM, or other crowdsourced data. These annotated images can, then, be used in remote sensing image classification tasks. Based on this method, we construct a worldwide large-scale benchmark for remote sensing image classification. This benchmark has large-scale geographical distribution and large total image number. It contains six categories with 35 sub-classes of more than 24,000 images of size 256×256 pixels. This classification system of ground objects is defined according to the national standard of land-use classification in China and is inspired by the hierarchy mechanism of ImageNet. Finally, we conduct numerous experiments to compare RSI-CB with the SAT-4, SAT-6, and UC-Merced data sets. The experiments show that RSI-CB is more suitable as a benchmark for remote sensing image classification tasks than other benchmarks in the big data era and has many potential applications.

## 1. Introduction

Image classification is a fundamental task in remote sensing image processing for which much meaningful research has been carried out [1,2,3,4,5,6]. Deep convolutional neural networks (DCNNs) have been considered as a breakthrough technology since AlexNet [7] achieved impressive results in the ImageNet Challenge [8] in 2012. DCNNs have brought computer vision applications into a new era of applications, such as image classification [7,9,10,11], face recognition [12,13,14,15,16], video analysis [17,18,19,20], and object detection [21,22,23,24,25,26]. Specifically, ResNet [9], which was developed by the Microsoft Asian Institute’s Visual Computing Group in 2015, achieved a 3.84% error rate in the ImageNet1000 Challenge, surpassing human performance on the data set for the first time [27]. Inspired by the success of using DCNN on natural image classification, remote sensing experts have introduced DCNN to remote sensing image classification and other recognition tasks [1,28,29,30,31,32], which is the current frontier and pinnacle of remote sensing image processing.

The key factors of the DCNN model’s success are its universal approximation ability, large-scale databases (such as ImageNet), and supercomputing ability when powered by graphics processing unit (GPU). DCNNs can learn effective feature representation from large-scale training data sets—such features are extremely important in computer vision tasks. Using large training sets is significant in two aspects: (1) Massive sets can help DCNNs, which are very complex and may have millions of parameters, to avoid over-fitting and obtain a more effective feature expression; and (2) large-scale sets can help to fill or approximate the entirety of the sample space as much as possible, which is an important factor for the generalization ability of DCNNs in some real-world applications.

However, the lack of a large-scale benchmark, which can be used as a standard to compare different image classification algorithm, makes the development of DCNNs in the remote sensing image field difficult. In recent years, several remote sensing image benchmarks have been built. In the following content, we will briefly review some of them.

The National Land Cover Database (NLCD) is a representative of early image classification benchmark. The latest version of NLCD is NLCD2016 [33]. It includes land cover data for the years 2016, 2013, 2011, 2008, 2006, 2004, and 2001. NLCD1992 [34] was the first U.S. land cover database with a 30 m spatial resolution, which characterizes objects in terms of pixels. NLCD2001 [35] added land cover data for three regions (i.e., Alaska, Hawaii, and Puerto Rico) based on NLCD1992. The concept of the database has been introduced into maps by incorporating the percentage of urban impervious surface and the percentage of forest cover, as well as improving the land cover classification method. NLCD2006 [36,37] is a 30 m spatial resolution database with images from Landsat7 and Landsat5, which inherited the NLCD2001. On this basis, NLCD2006 added land cover and impervious surface change data between 2001 and 2006. NLCD2011 [38] provides a decade of land cover change for the conterminous U.S. (CONUS) over three time periods. NLCD2016 improved the look and feel of NLCD by integrating pixel-based and shape-based classification and new ancillary data layers. NLCD characterizes objects in pixels. These objects include the open category of water, shrubs, grassland, and so on. Identifying small objects is very difficult, due to the benchmark’s low spatial resolution. Therefore, NLCD is specially applicable to general land-use identification.

UC-Merced [39] is a famous satellite imagery database for scene classification. It is made up of urban area images from the United States Geological Survey (USGS). UC-Merced includes 2100 images sized 256×256 pixels with a 0.3 m spatial resolution. It has 21 categories, including overpass, plane, baseball, beach, building, dense residential, forest, freeway, golf course, harbor, and other objects. Each type of land has 100 images. UC-Merced has geographic objects of different textures, colors, and shapes. It has a high spatial resolution and has been widely used in remote sensing image classification [40,41,42,43,44,45]. In this database, however, the number of images in each category is small and thus not suitable for the distribution of overall characteristics for actual geographic objects.

SAT-4 and SAT-6 are another two important benchmarks for remote sensing classification, which were collected by the National Agriculture Imagery Program (NAIP) [46]. The aerial images of these benchmarks were obtained from rural, urban, densely forested, mountain, water, and agricultural areas in California. SAT-4 and SAT-6 consist of six categories—bare soil, vegetation, grassland, road, house, and waterbody—which cannot achieve the purpose of remote sensing image recognition in practical applications. SAT-4 and SAT-6 have 500,000 and 405,000 images, respectively. The image patches are sized 28×28 pixels, which is very small. Hence, the details of the internal image are insufficient. The small block size cannot reflect the complex distribution of features completely, and the image may contain only the local features of objects.

Terrapattern [47], which was constructed at the Carnegie Mellon University, can search for similar objects, according to given objects within a specified area (currently including New York, San Francisco, Pittsburgh, Detroit, Miami, Austin, and Berlin, Germany). The search results can be marked on the map, which provides a new method for constructing a remote sensing image data set.

The aerial image data set (AID) [48] is a remote sensing image data set built by Wuhan University. Its images were taken from Google Earth. It contains 10,000 images with 30 categories, where each category contains approximately 330 image blocks with spatial resolutions ranging from 0.5 to 8 m per pixel. The image blocks used are sized 600×600 pixels, due to the different resolutions of the features. AID has three significant features: (1) It has strong diversity in imaging angles, shapes, sizes, colors, surrounding environments, and so on; (2) the difference between several scene objects, such as schools and intensive residential areas, is small, and these factors potentially improve the difficulty of classification; (3) AID is large-scale, compared with other existing data sets. However, AID has two disadvantages: First, AID does not have an efficient database construction method, and has no set forming system for developing database categories with geographical and practical values. Second, the AID database scale is not large enough for high-resolution remote sensing applications.

NWPU-RESISC45 [1], which was built by the Northwestern Polytechnic University, was taken from Google Earth with a spatial resolution of 0.2–30 m per pixel. It has 45 categories and 31,500 256×256 pixels images, which are mainly used for remote sensing image scene classification. In addition to continuously maintaining object diversity based on AID, the NWPU-RESISC45 data set is characterized by an improved image scale. Common object types were considered in the selection of categories and through “OBIA,” “GEOBIA,” “geographic image retrieval,” or other keyword searches to determine the final 45 categories, which indicates that the NWPU-RESISC45 data set pays more attention to objects with geographical significance and application value. However, its disadvantage lies in the inefficiency of the NWPU-RESISC45 data set in constructing a remote sensing image database, particularly in not using existing geographic crowdsourced data as auxiliary information.

A large-scale benchmark is critical for remote sensing experts, in order to improve their models and algorithms, as deep learning methods have come to govern imaging-related tasks in the big data era. However, building high-quality and large-scale benchmarks is challenging, because (1) a remote sensing image has numerous complex objects, rather than simple objects of one nature; (2) the objects in remote sensing are at a global scale; and (3) remote sensing images are affected by several factors, such as the camera’s perspective, weather conditions, and solar altitude angle.

This paper presents a method based on crowdsourced geographic data to build a remote sensing image benchmark. Crowdsourced geographic data are open geospatial data provided by the public or related institutions through the Internet [49]. Crowdsourced geographic data sources are large, rich in information, diverse in categories, low cost, and are in real time [50]. Hence, using crowdsourced geographic data to annotate remote sensing images provides a potential approach for building high-quality and large-scale benchmarks. We select points of interest (POIs) from the Open StreetMap (OSM) [51] in different locations. Then, we align the POIs with different temporal remote sensing images downloaded from the Google Earth and Bing Map servers, according to the geo-coordinate. We call this the remote sensing image classification benchmark (RSI-CB( This benchmark can be downloaded from https://github.com/lehaifeng/RSI-CB)), which uses POIs from different countries. According to the POI distribution density, we select high-density areas in major cities around the world. Other countries and their regions that have strict hierarchical systems are also included. The remote sensing images are downloaded from Google Earth and Bing Maps with 0.22–3 m spatial resolutions. RSI-CB contains six categories with 35 subclasses of more than 24,000 images. Inspired by ImageNet [8], we combine the hierarchical grading mechanism of the Chinese land-use classification standard [52] to satisfy the diversity and comprehensive requirements of the object class further, in order to construct a generalized benchmark. Currently, RSI-CB has six categories—agricultural land, construction land and facilities, transportation and facilities, water and water conservancy facilities, woodland, and other land uses—and each of them has several sub-classes.

The main contributions of this paper are as follows:

1. We propose a crowdsourced data-based method to build RSI-CB. The crowdsourced data in our method is a high-precision supervisor. Traditional methods require a significant amount of manual work; thus, they are less efficient and time-consuming. Using crowdsourced data as a supervisor facilitates machine self-learning through the Internet. Moreover, the size of the benchmark sample could be vast, both in amount and variety. In addition, crowdsourced data are basic data sources in the big data era and are updated rapidly. Therefore, a remote sensing benchmark constructed using crowdsourced data may continue to expand, in terms of the diversity, quantity, and robustness of samples. Consequently, our method can potentially realize weak supervised learning further for remote sensing images.
2. Based on the above method, we construct a global-scale RSI-CB. We build a hierarchical grading object category system. The system contains six categories with 35 subclasses. With diverse objects, rich categories, and a large number of images, this data set can be used to effectively develop new data-driven algorithms and further improve the state-of-the-art methods
3. We conduct various experiments to compare RSI-CB with SAT-4, SAT-6, and UC-Merced data sets on handcrafted features, such as scale-invariant feature transform (SIFT) [53], color histogram indexing (CH) [54], local binary patterns (LBP) [55], GIST [56], and classical DCNN models, such as AlexNet [7], VGGNet [10], GoogLeNet [57], and ResNet [9]. In addition, we demonstrate that DCNN models trained by RSI-CB have good performance when transferred to the UC-Merced data sets, and have good generalization ability. The experiments show that RSI-CB is a more suitable benchmark for remote sensing image classification than the other existing benchmarks in the big data era and has many potential applications.

The rest of this paper is organized as follows. Section 2 describes the basic requirements for a remote sensing image benchmark for deep learning and the crowdsourced data-based method for building a remote sensing image benchmark. Section 3 presents an analysis of the properties of RSI-CB, in terms of geographical distribution, category hierarchy, and statistical distribution, as well as a comparison of RSI-CB with other remote sensing benchmarks. Section 4 discusses the results of the tests on classification performance using handcrafted feature methods and classic DCNN models on RSI-CB and other benchmarks. Section 5 concludes the paper and presents future research directions.

## 2. Remote Sensing Image Labeling Method Based on Crowdsourced Data

### 2.1. Basic Requirements for Remote Sensing Image Benchmark Using Deep Learning

Deep learning models, such as DCNN, have seen significant breakthroughs in various tasks, such as image tracking and scene understanding. DCNN models are very complex and have millions of parameters; hence, they are easily overfitted on small benchmarks [58]. From the learning theory perspective, DCNN models have high VC dimension (an abstract representation of model’s learning ability), which results in complex and diverse samples [58]. From the optimization algorithm perspective, DCNNs often employ the stochastic gradient descent (SGD) algorithm [58]. The SGD algorithm often results in very little change in each neuron’s parameter when a lower learning rate is used and, hence, the sample complexity is limited. Therefore, we argue that the RSI-CB for DCNN models should be governed by the following factors:

(1) Scale of benchmark
  DCNN models have a strong learning ability and the ability to approximate any function, which can be combined with large data sets to better describe the inherent characteristics of the distribution of large data. Moreover, the image classification effect is related to the depth and width of the network model, which corresponds to network complexity. A more complex network model indicates more training parameters, which require more image training samples. In addition, when the sample data is insufficient, sampling error cannot be ignored, even when the network is simple. When sample features do not fit well with the distribution of actual features, the knowledge learned by the model is insufficient as well, leading to unsatisfactory robustness and generalization ability.
(2) Object diversity
  A strong distinction exists within classes. In constructing large-scale data sets, the data should be representative of the classes, causing the learning model to learn not only the unique characteristics, but the general ones as well. Therefore, different images of the same type of object (such as aircraft) should have different sizes, shapes, perspectives, backgrounds, light intensities, colors, and other conditions that diversify the objects within the class. Our images come from Google Earth and Bing Maps, so the images come from different sensors. To improve the class diversity further, the selection within categories should be based on the diversity requirements mentioned above.
(3) Category differences
  In addition to the idea that massive representative training data can help to learn more visual features, the differences between classes is also a determinant of image classification accuracy. In data set construction, if the differences between classes are large, the probability of independent feature interval distributions in each category is higher, whereas similarity between classes leads to a higher overlapping ratio of various features. Therefore, a feasible method is to increase the number of images in these categories, which can cause the inter-class feature response interval to have a higher probability of being an independent distribution, further improving the accuracy of image classification.

According to these requirements, the construction principles for RSI-CB are as follows:

- Each category must be rich with data. RSI-CB includes approximately 690 patches per category.
- The method for selecting object categories combines the category system of the Chinese national land-use classification standard and the hierarchy mechanism of ImageNet. The level of each category aims to increase the diversity and comprehensiveness of the benchmark. The data set is arranged in a two-level tree: with 35 leaf nodes (sub-classes) connected to six parent nodes (parent classes).
- The main body of the object must be easy to identify, which can avoid semantic divergence in images.
- Each class has different imaging angles, sizes, shapes, and colors, in order to increase sample diversity, which can improve the model generalization performance and robustness.
- The RSI-CB selects images that come from major cities worldwide and their surrounding areas, considering the balance of spatial distribution for the selected images.

### 2.2. Remote Sensing Image Labeling Method Based on Crowdsourced Geographic Data

Crowdsourced geographic data representation is achieved through GPS route data (such as the OSM data used in this paper), map data edited by user collaboration plans (such as Wikimedia data), various social network data (such as Twitter and Facebook), and POI data where users have checked in. These data need to be processed, in order to form standardized geographic information. The crowdsourced geographic data labeled by non-professional people are highly real time, have high spread speed, are rich in information, are low-cost, and are large in quantity, compared with those from traditional geographic information collection and updating methods. Thus, crowdsourced data has become a key research focus. However, the imbalance of quality and density distribution, redundant and incomplete data, and a lack of uniform norms and other defects for such data are the main drawbacks in using crowdsourcing [59].

The construction of the RSI-CB mainly uses two types of data, namely POI in OSM and remote sensing image data. OSM elements include layers, nodes, and ways. In the POI data, we define the positions of points for their spatial location, which are stored as latitude and longitude together with attribute information. The attribute information of POI data is shown in Table 1. POIs are selected from different countries and regions worldwide. Remote sensing images are obtained from Google Earth and Bing Maps. The spatial resolution we used ranged from 0.22–3 m per pixel.

**Figure 1:**
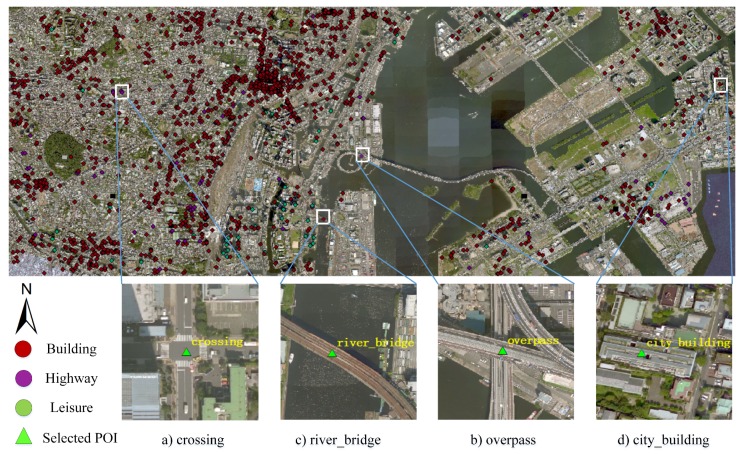
Superposition effect of the POI data and image. The four small maps are the categories of crossing, overpass, river_bridge, and city_building, respectively, which are used as the center points for cropping remote sensing images (the large image is part of the Shanghai area, and its spatial resolution is sub meter).

The core ideas for constructing RSI-CB can be summarized as follows:

- Construct a registration overlay of high-resolution remote sensing images and POI data, making sure that the actual targets in images correspond correctly with the POI.The superposition effect of POI data and image are shown in Figure 1.
- POI data screening: This screening includes the deletion of wrong annotations, removal of non-conformal objects which indicate no intersection with the Chinese national land-use classification standards (see Section 3.2), and deletion of objects which are not obvious (see below for a detailed discussion).
- Cropping fixed-size remote sensing image blocks according to the POI data, traversing all POI, taking each POI as the center to crop remote sensing images with fixed sizes of 256×256 pixels, and then integrating the same category of image blocks to build the final benchmark.

The process flow of constructing RSI-CB can be briefly described as follows:

1. Acquiring raw POI and remote sensing image data.
2. Filtering the POI data, according to the intersection of the Chinese land classification standard and POI data.
3. Based on the geographical coordinate information of each POI, taking each of POI as a center and cutting the image blocks to a preset size from the remote sensing image.
4. Applying the feature type of each POI as the label of the image block.

However, the uneven coverage of POI data results in different density distributions as well as frequent category updates and incorrect phenomenon labeling by artificial methods. Therefore, using POI to construct a data set leads to two obvious problems. At this stage, we solve these two problems manually.

**Figure 2:**
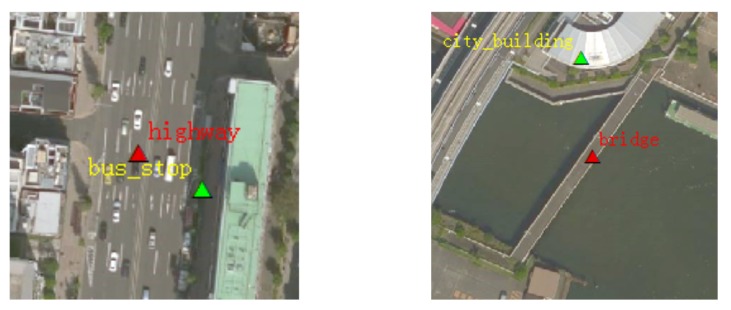
Multi-labeling in a small candidate area. Red annotations are the retention categories, and the yellow annotations should be deleted. The left side of the candidate area shows “highway”; “bus_stop”, and “bus_stop” should be removed, due to their small size and unclear recognition. The right side of the candidate area shows “bridge”; “city_building” should be removed due to its lack of salience and as it is smaller than the bridge. This may not be the case for another image, where city_building may be dominant.

**Figure 3:**
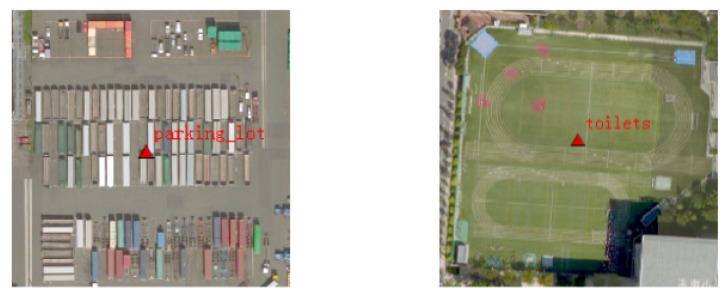
Candidate area error labeling phenomenon. The red label is the wrong category for the images. The left area should be labeled as “container”, not “parking_lot”. The right area should be labeled “artificial_grassland”, not “toilets”.

1. Several points stacked in a small area, with these points as the centers of image blocks (256×256 pixels) with high overlapping ratios. For this phenomenon, data screening follows the principle that the area of such a category should be dominant and its recognition degree high. ( Figure 2).
2. For some POI data, the names of the objects are not exactly matched with those of the remote sensing image objects. For this phenomenon, we delete the POI data directly. (Figure 3).

## 3. RSI-CB Statistical Analysis

### 3.1. Geographical Distribution of RSI-CB

**Figure 4:**
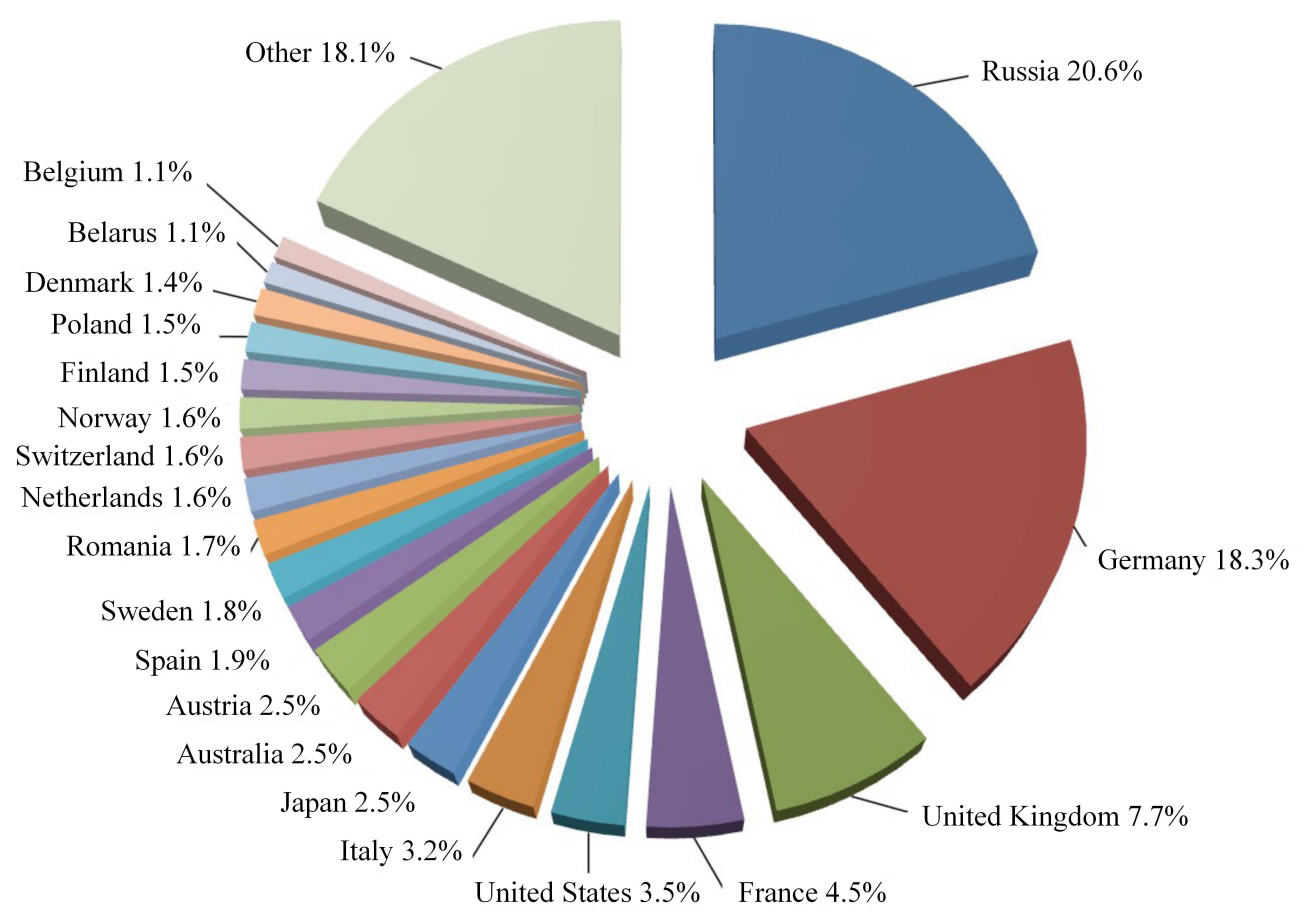
Distribution of POI data worldwide.

OSM is a collaborative crowdsourced map program, which aims to create a free map. Anyone worldwide can contribute to OSM. Volunteers from 230 institutions and countries upload data to OSM every day. Hence, the OSM data are large-scale and widely geographically distributed. These data can be used as annotation sources for the sustainable labeling of global remote sensing images. Figure 4 shows the distribution of POI data worldwide. Russia has POI coverage of up to 21%, due to its extensive land area and local taxi companies and travel agencies contributing their data. Germany follows with 18.3%. The U.K., France, the U.S., and other countries also have a greater proportion. Hence, we can yield balanced geo-labels for remote sensing images by controlling the ratio of POIs from different regions worldwide.

Section 2.1 presented the requirements for constructing our database. Hence, to train a more robust and generalized classification model—besides the requirements that the database itself should be large—another key point is that it also must make sense. There are four factors to be considered: First, the database is designed to be used by researchers around the world and, so, the selected areas should contain as many cities as possible, with the characteristics of regions belonging to different categories; second, to meet the requirements of sample diversity, the images of RSI-CB must come from various regions of the world, in categories which have different shapes, sizes, and color characteristics, but also have different intensities of light, shadow conditions, and backgrounds; third, according to the distribution of the crowdsourced data, the selected cities should have large data distributions; and fourth, the remote sensing images of the selected regions should have high spatial resolution. Taking into account the above four factors, we selected POI data distributed in major countries, cities, and regions around the world, such as Beijing, Shanghai, New York, Washington D.C., London, and so on.

**Figure 5:**
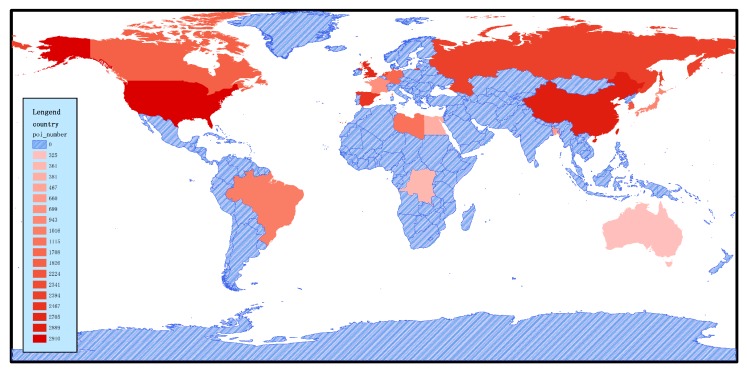
Geographical location and quantity distribution of selected POI. Darker areas indicate greater amounts of POI data selected (the number in the legend indicates the number of POIs selected in the region).

Figure 5 shows the geographical and quantitative distribution of the selected POI data in major countries and regions. The darker areas indicate a greater amount of POI data, and the blue areas indicates those that RSI-CB has not yet selected. Among them, the U.S., China, and the U.K. feature the largest amount of data, including the categories of agricultural land, construction land and facilities, transportation, and facilities, as well as other large categories. At the same time, the Brazilian woodland, Egyptian desert, and Canadian snow have different distributions.

### 3.2. Hierarchy for RSI-CB Categories

The purpose of remote sensing image classification is to extract important and significant feature types from images [60]. However, given the complexity of the background environments in remote sensing images, as well as the diversity of the feature categories and other external conditions of the objects, such as shape, size, color, and other factors, determining the categories of objects, then classifying and ensuring the rationality and practical significance of the categories are key points in constructing a remote sensing image benchmark.

Hence, to meet the diversity within and among classes, we conducted two methods to establish our classification criteria:

1. We analyzed the existing attributes of OSM data and the Chinese classification criteria of land use and selected the common categories among them as our preferred classes.
2. We deleted the data that do not meet the basic requirements of the benchmark, as well as the using the principles for constructing the RSI-CB in Section 2.1.

Table 2 shows the correspondence between the Chinese land-use classification standard and the OSM categories. The four categories “Residential”, “Transportation”, “Leisure”, and “Natural resources-related” had the largest degree of overlap. According to our database establishment standard, we deleted “Leisure”, as the “Leisure” category is not common in remote sensing image scenes. “Natural resources-related” in the Chinese land classification standard includes “agricultural land”, “woodland”, and “water”. Hence, we finally chose six categories: construction and facilities, transportation and facilities, agricultural land, water and water conservation facilities, woodland, and other land.

**Figure 6:**
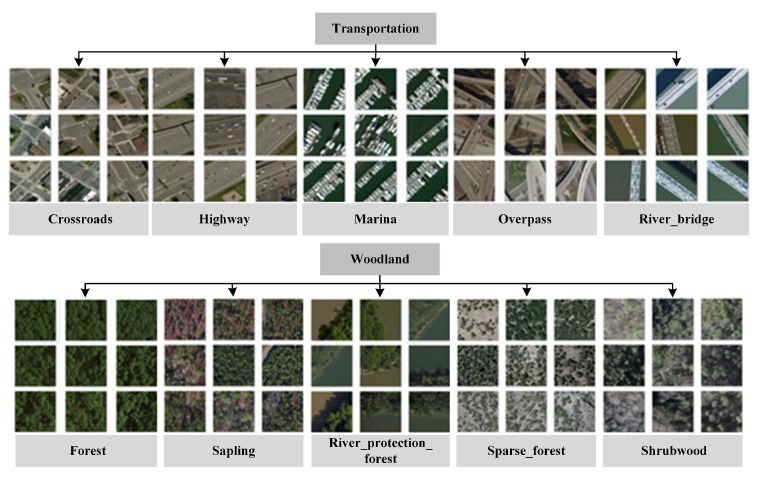
Root and leaf nodes of remote sensing image classification benchmark (RSI-CB). The category of transport corresponds to the sub-classes of crossroads, highway, marinas, overpass, river_bridge, and others. The next figure shows the category of woodland, with sub-classes corresponding to forest, sapling, river_protection_forest, sparse_forest, shrub wood, and others.

On the basis of the classification of different levels in the Chinese land classification standard, we selected three sub-classes under agricultural land, seven under woodland, eight under transportation and facility, seven under water area and facility, six under construction land and facility, and four under other land. Figure 6 shows some of these sub-classes.

### 3.3. Distribution Characteristics of RSI-CB

**Figure 7:**
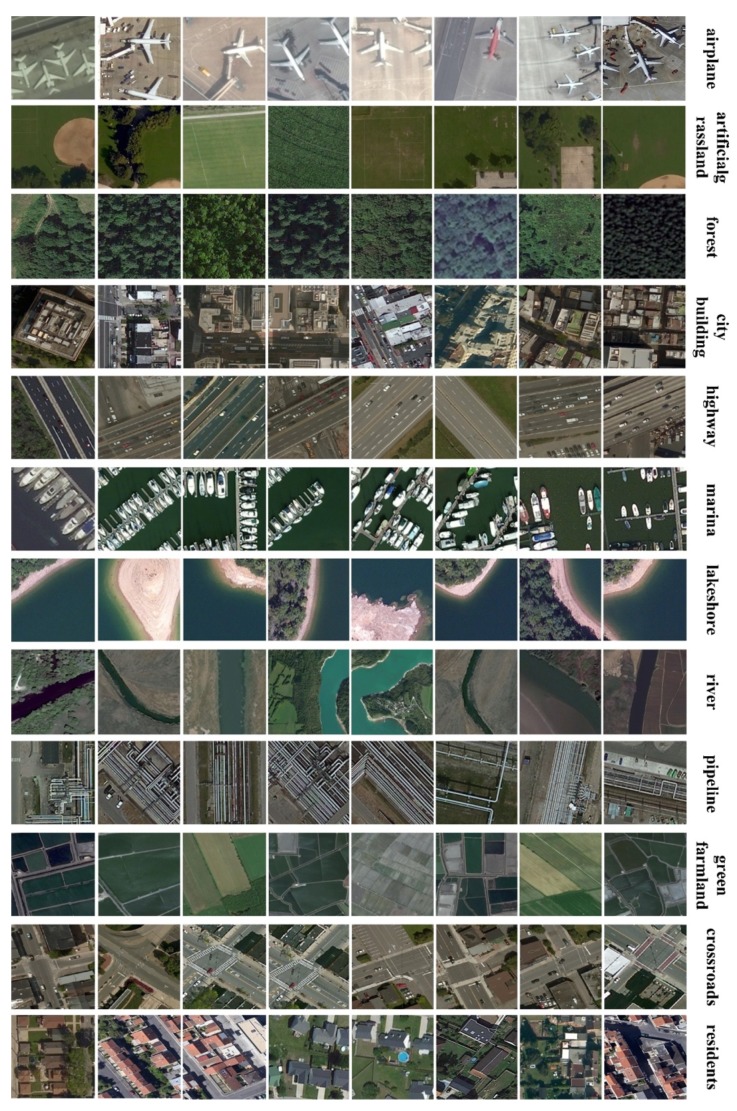
Samples of RSI-CB.

We finally built constructed large categories—agricultural land, construction and facilities, transportation and facilities, water and water conservancy facilities, woodland, and other land—according to Section 2.1 and Section 3.2. Our RSI-CB benchmark has 35 sub-categories with approximately 24,000 images, with an average of approximately 690 images per class. The large categories correspond to its subclasses, as shown in Table 3. The large categories of transportation and facilities, woodland, and water and water conservancy facilities contain more subcategories. The distribution of each category is shown in Table 4. Figure 7 show some samples of the RSI-CB benchmarks.

### 3.4. Comparison of RSI-CB with Some Remote Sensing Data Sets

(1) Spatial resolutionRSI-CB has a higher spatial resolution than other existing data sets, which can be seen clearly from the image memory size: each image in AID is approximately 20–80 kb, with an image size of 600×600 pixels; and the images of the NWPU-RESISC45 data set are approximately 10–20 kb, with an image size of 256×256 pixels In this study, each image was almost 193 kb for RSI-CB. This high spatial resolution means more information detail and more comprehensive characteristic object information, which is useful for object recognition.
(2) Scale of benchmark
  RSI-CB is a larger-scale benchmark than any of the other databases, having approximately 24,000 images distributed in 35 categories (with approximately 690 images per category); compared to the relatively recent large-scale benchmark AID, in which there are 10,000 images distributed in 30 categories (with about 330 images per category).
(3) Construction model
  Unlike the previous database construction model, RSI-CB makes full use of crowdsourced geographic data, which has three main contributions to remote sensing image data set construction. First, we use the crowdsourced data for a high-efficiency and high-quality method to build a remote sensing benchmark, as well as providing the possibility of continued expansion, in terms of the diversity, richness, and scale of the benchmark. Second, we reflect the geographical significance of each geographic entity itself by constructing remote sensing data set with crowdsourced geographical data and making use of the criteria of the Chinese land-use classification in combination with the ImageNet hierarchical grading mechanism. Finally, we can use computers to achieve self-learning and learn the overall characteristics of objects according to massive crowdsourced data in the future, which can assist in automatic labeling and recognition purposes to further understand the images.
(4) Diversity of benchmark Database diversity metrics can be divided into intra-class diversity and inter-class diversity. Diversity can be measured by similarity: for the same class, if the similarity is higher, then the diversity is lower, and similarly for intra-class diversity. Therefore, we use the image similarity measure to verify the intensity of the image diversity, using the Bhattacharyya distance [61] to calculate the difference between vectors to reflect the degree of image similarity. In the statistical theory, the Bhattacharyya distance is used to measure the similarity of two discrete or continuous probability distributions as an approximation of the overlap between two statistical samples.

**Figure 8:**
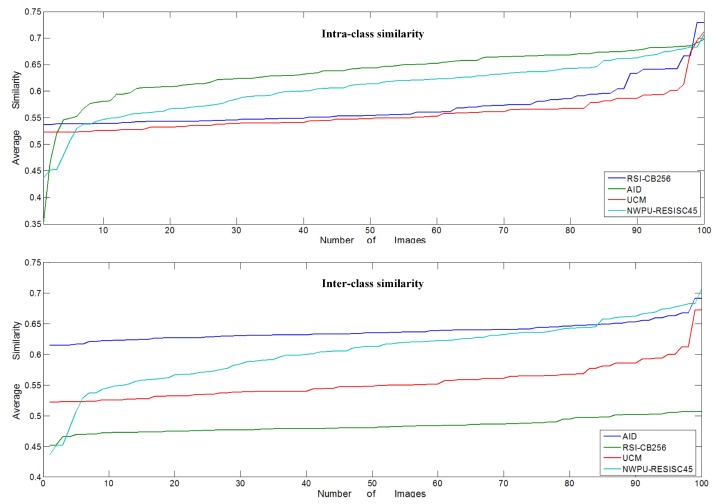
Intra-class similarity and inter-class similarity among the four benchmarks.

We randomly selected the sample categories ‘river’ and ‘parking’ among the four benchmarks, with 100 images for each category based on the number limit of the UC-Merced benchmark to calculate the inter- and intra-class similarity between the categories. We calculated the similarity between each of the images with any other image, then averaged the values. Figure 8 shows the results of the similarity measurement among the four benchmarks; for the inter-class similarity measurement, it shows the average similarity of ’river’ and ‘parking’. As the figure shows, RSI-CB256 had lower inter-class similarity than the existing, relatively large benchmarks AID and NWPU-RESISC45, but was a little higher than UC-Merced. However, RSI-CB256 obtained the lowest intra-class similarity, compared to the other benchmarks, which was in the range of 0.45–0.51.

**Figure 9:**
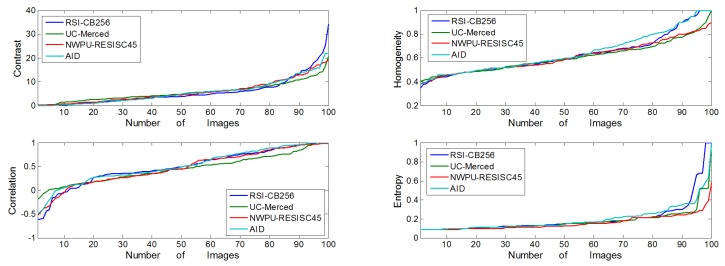
Comparison of image texture features among the four benchmarks using GLCM.

The co-occurrence matrix is defined by the joint probability density of the pixels at two positions, which not only reflects the distribution characteristics of the luminance but also the position distribution characteristic between pixels with the same or similar brightness, which forms the basis for defining a set of texture features. In order to be more intuitive in describing the texture of the co-occurrence matrix, we described the following four parameters separately: (a) Contrast, which reflects the image sharpness and texture of the extent of the groove depth, the deeper texture groove means the greater contrast and the higher clarity; (b) Homogeneity, which measures the degree of local changes in image texture, wherein a larger value means the image textures of different regions lack changes; (c) Correlation, which reflects the local gray image correlation, when the matrix pixel values are equal, the correlation value is large; to the contrary, if the matrix pixel values vary widely, the correlation value is small; and (d) Entropy, which represents the complexity of the information within an image. Figure 9 shows the values of the four parameters mentioned above among the four benchmarks; they have almost the same trend among them, but the contrast, correlation, and the entropy information of RSI-CB256 were relatively large, indicating that the RSI-CB256 image is clearer for recognizing objects and features. Furthermore, the high entropy suggests more complex images which, to some extent, reflects stronger diversity.

Table 5 shows the comparison of several important factors for benchmarking, including the number of images and categories, spatial resolution, and size of images. The SAT data set had the largest number of images, SAT-4 had an average of more than 80,000, and SAT-6 had nearly 70,000 images per category, but the obvious drawback was that only six categories were used and the images were too small, as image size was sacrificed to obtain more images. NWPU-RESISC45 had 45 categories more than RSI-CB. However, the difference between them was that RSI-CB had more for object classification and NWPU-RESISC45 had more for scene classification, due to different spatial resolutions and image sizes. Besides that, the most important difference is that we constructed RSI-CB in a highly efficient way, which can be updated continuously with new crowdsourced data.

In general, RSI-CB has the advantages of spatial resolution, scale of quantity, and a novel means of constructing a database, compared to other well-designed benchmarks. However, the construction of RSI-CB lies not only in the meaning of the database itself, but also in the crowdsourced data-based method for its potential application value in weak-supervised learning to realize the automatic marking and error correction of data.

## 4. Experimental Analysis

### 4.1. Test Methods

**Figure 10:**
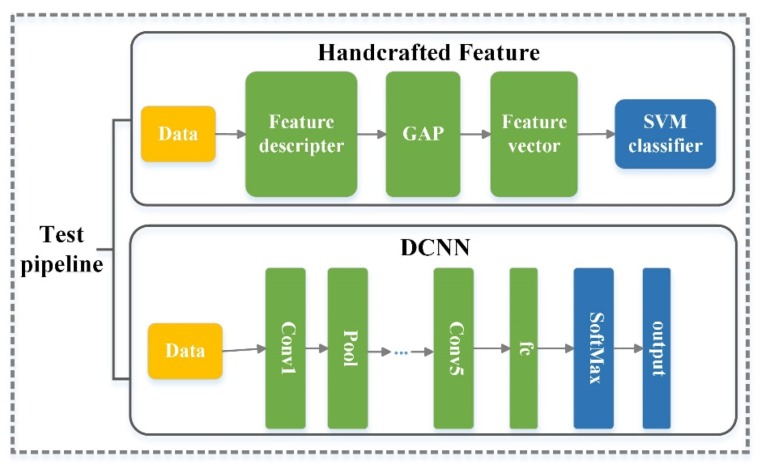
Test pipeline for RSI-CB (“Conv” means convolution layer, “Pool” means pooling layer, “fc” means fully connected layer, and “SoftMax” is an activation function).

(1) Model selection: handcrafted feature models versus DCNN (learning feature) models
  We used traditional handcrafted features and DCNNs based on end-to-end learning features as the test pipeline to test the performance of different methods on RSI-CB. As shown in Figure 10, for handcrafted features, we used SIFT [53], Color Histograms (CH), LBP, and GIST as the description operators and global mean pooling to construct the eigenvectors for these methods. Finally, the SVM model was employed to classify the images. For the end-to-end learning features, we used AlexNet [7], VGG-16 [10], GoogLeNet [57], and ResNet [9] to train RSI-CB. Figure 10 shows the test pipeline for RSI-CB. The top half of the figure introduces the test pipeline with handcrafted features. The bottom half of the figure shows the test pipeline with DCNN.
(2) Training methods
  We trained DCNN models from scratch, rather than fine-tuning them, for the following reasons. First, considering the slight similarity between RSI-CB and ImageNet, the former uses satellite imagery and the latter uses a natural imagery. The second reason is to define our own network structure. Third, the scale of RSI-CB is relatively large. The last reason is that it is convenient for small-scale remote sensing image data sets to fine-tune.
(3) Testing for model transfer performance
  Transfer performance testing was based on the RSI-CB training model, which tests the model’s ability to identify other databases. We selected UC-Merced for the test database. We chose the lighter AlexNet-Conv3 model, considering the small scale of UC-Merced.

### 4.2. Data Organization

Data organization has three main points: selection of the training, validation, and test sets for RSI-CB; data augmentation; and data organization for model transfer performance.

1. Selecting data randomly: The training, validation, and test sets are randomly selected according to a certain proportion, and we disrupt labeling to further reflect the randomness of the data and objectivity.
2. Data augmentation: We expand all RSI-CB data for each image by cutting a fixed-size patch from the center and upper-left, upper-right, lower-left, and lower-right corners of each image, and then flip them before using as input to the DCNN. Thus, the original data is expanded 10-fold.
3. Data organization for model transfer performance: We test whether the RSI-CB training model can transfer to other data sets and the strength of its ability to transfer. We selected 13 categories which were common to UC-Merced and RSI-CB as experimental data. The size of these two types of data was 256×256 pixels, as UC-Merced has only 100 images in each class. We selected 1300 images for RSI-CB and UC-Merced as the test set.

### 4.3. Parameter Settings

**Figure 11:**
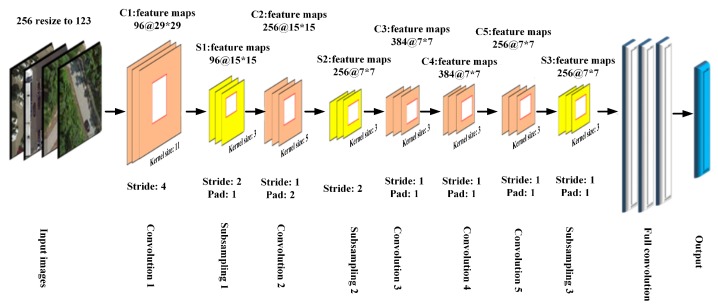
AlexNet model parameter settings in RSI-CB ("96@29*29"means there are 96 feature maps of 29×29 pixels. The “Stride” and “Pad” are the parameter of AlexNet).

(1) Handcrafted features
  We refer to the method of Reference [48], which used four low-level features—namely, SIFT, LBP, CH, and GIST—as feature description operators. For the SIFT descriptor operator, we used a 16×16 fixed-size grids with a step of eight pixels to extract all descriptive operators in the gray image. Each dimension describing the operator used the average pooling method to finally obtain the 128-dimensional image feature. For LBP, we used the usual eight directions to obtain the binary value and converted the 8-bit binary value to a decimal value for each pixel in the grayscale image. Finally, we obtained the LBP feature by calculating the frequencies of the 256 gray levels. For CH, we used the RGB color space directly and quantized each channel into 32 bins. Therefore, we obtained the features of images by connecting the statistical histogram in each channel. For GIST, we used the original parameter settings from Reference [56] directly, using four scales, eight directions, and a 4×4 spatial grid for pooling. Finally, we obtained a 512 (4×8×4×4) dimensional eigenvector.
(2) DCNN models
  We retained most of the default parameters to train the DCNN and fine-tune the learning rate and batch size. Our model converged better, although we made concessions in terms of the computational time and convergence rate. In addition, the vibration of the loss function value was smaller, which was beneficial in improving the performance of our model. In the RSI-CB test, we implemented a slight adjustment in the network due to the input size constraints of the network and adjusted the padding for the convolution and pooling layers. We did not warp images, as this affects the real information of the images, to some extent. Figure 11 shows the AlexNet model parameter settings in RSI-CB.

### 4.4. Evaluation Methods

We used two common methods as evaluation indices for the training model: overall accuracy (OA) and confusion matrix [62].

1. OA refers to the ratio of the number of categories that are correctly classified to the total number of categories. The OA value can provide a good characterization of the overall classification accuracy. However, when the categories are extremely imbalanced, the OA value is greatly affected by categories with more images.
2. The confusion matrix can visually reflect the classification accuracy of each type of object. We can clearly determine the correct and wrong classifications in each category of each row. For better visualization, we use a thermodynamic chart to display the confusion matrix.

### 4.5. Experimental Results

The experimental results are divided into three parts: classification results based on handcrafted features and DCNN and model transfer ability test results. We used different test methods in the first two parts of the results to evaluate our benchmark and differentiate the tests in the UC-Merced, SAT-6, and SAT-4 data sets to compare their performance. For the transfer ability test, we extracted 13 common categories of RSI-CB and UC-Merced with 100 images per category and named them RSI-CB-13 and UC-Merced-13, respectively. Then, we trained AlexNet-Conv3 with the remaining images in RSI-CB-13. Finally, we validated the classification ability of the model on UC-Merced-13.

#### 4.5.1. Classification Results Based on Handcrafted Features

We used the UC-Merced and RSI-CB benchmarks with a uniform size of 256×256 pixels as experimental data to ensure the fairness of results. We tested the data 10 times and took the mean and standard deviation as the results.

Table 6 shows the means and standard deviations of the OA, based on the handcrafted features of the UC-Merced and RSI-CB benchmarks. The values in brackets refer to the ratio of the training set to the total number of data. The overall test results using the SIFT method for RSI-CB were relatively low, indicating that SIFT was inadequate in representing the characteristics of our remote sensing images. The best results were achieved using the CH method on RSI-CB, with an accuracy of more than 80%. However, its result was not the best on UC-Merced, due to the following two reasons: We had a clear advantage in the number of images and there were some differences between RSI-CB and UC-Merced, which are mainly reflected in the diversities and complexities of the databases.

In addition, the experimental results show that no universal recognition algorithm exists for different data sets, and that test results improved significantly when the training set size increased, which indicates that the amount of data is still a key factor in improving recognition accuracy.

**Figure 12:**
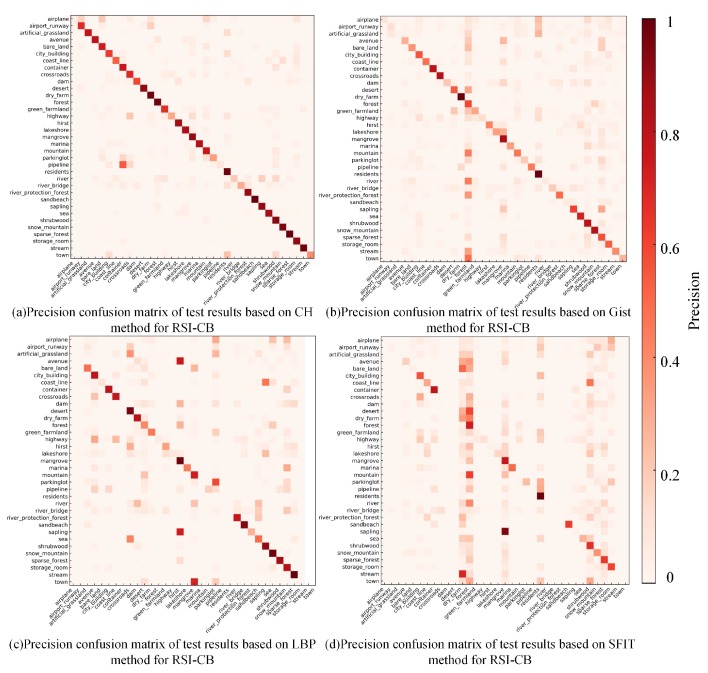
Precision confusion matrix of test results based on handcrafted features for RSI-CB.

Figure 12 shows the precision confusion matrix of the test results using handcrafted features on the RSI-CB benchmark. From the precision confusion matrix above, the classification precision of a single category differed in various methods, and each category of its classification performance was consistent with OA. SIFT was still inadequate for classifying most categories. The classification precision of nearly half of the categories based on CH is more than 0.8, and LBP also achieved good results. However, these methods performed poorly for some categories (e.g., airplane and parking lot) due to the small differences between category features and the large overlapping ratio of the inter-class feature distribution. Furthermore, there were richer features in some categories. Consequently, handcrafted methods are usually used for describing low-level features and cannot fully describe the distribution of features.

**Figure 13:**
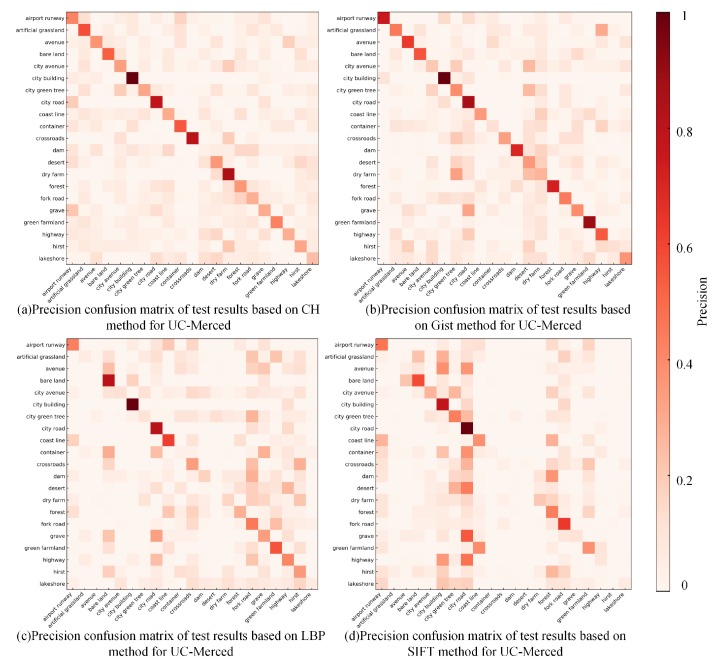
Precision confusion matrix of test results based on the handcrafted features for UC-Merced.

The performance based on handcrafted features on UC-Merced, as shown in Figure 13, was much worse than the test results in RSI-CB, where only few categories reached a precision of 0.8. The main reason for this lies in the obvious scale advantage of the RSI-CB benchmark. In addition, RSI-CB is an object-centered benchmark for most categories, whereas UC-Merced is based on complex scenes for most categories. Each image contains more complex information and robust visual features.

In summary, methods based on color, texture, and other handcrafted features cannot perform well in complex object feature recognition, as manually designing low-level visual features and obtaining an optimal eigenvalue and expressing it appropriately both directly affect the precision of classification recognition.

#### 4.5.2. Classification Results Based on DCNN

We tested RSI-CB and compared it with other remote sensing data sets, based on the DCNN method. We used the AlexNet-Conv3 network with only three convolutions to test SAT-6 and SAT-4, due to their small size (28×28 pixels).

Table 7 describes the overall performance of different models based on DCNN for the different data sets. The test results of the models for RSI-CB were more than 90%. The classification accuracy of the GoogLeNet results was lower than that of the network structure of GoogLeNet, as the wide range of macro performance in objects for RSI-CB does not require high-precision features to represent, which may be slightly different from that in natural images. The recognition accuracy of GoogLeNet in RSI-CB was less than AlexNet, which contradicts the findings mentioned above. The overall recognition performance of the models does not rely only on the network depth, but also depends on different network structures and the data itself. VGGNet performed unexpectedly better than the top-1 of ResNet in natural image recognition, which further shows that the performance of a model is undoubtedly related to the benchmark itself and the difference between remote sensing and natural image databases. The results of our benchmark were greatly improved, due to the obvious advantage of data scale compared with the UC-Merced. The SAT-6 results were highly satisfactory. This is probably due to its small number of categories and low complexity of images with many similar features, which make it easy to obtain excellent performance.

**Figure 14:**
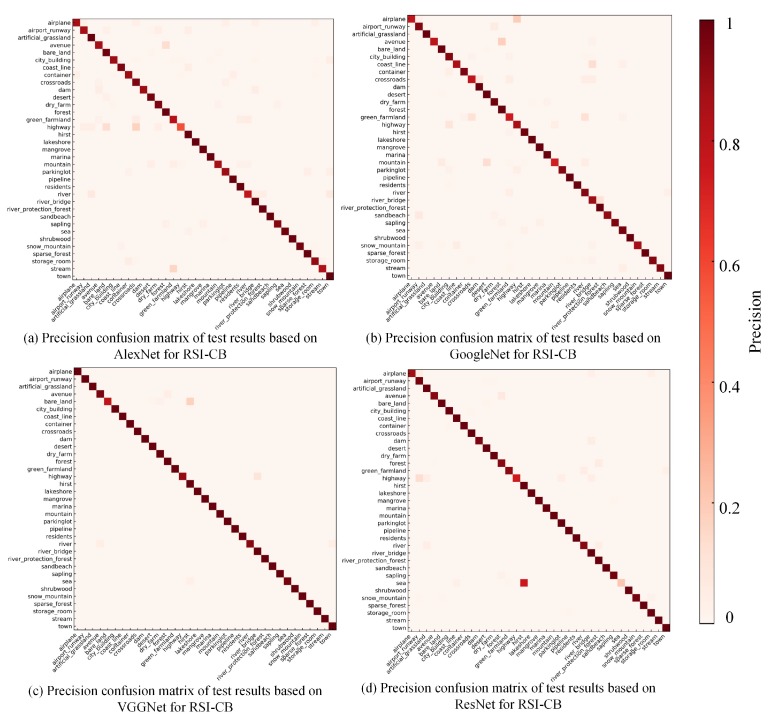
Precision confusion matrix of test results based on DCNN models for RSI-CB.

**Figure 15:**
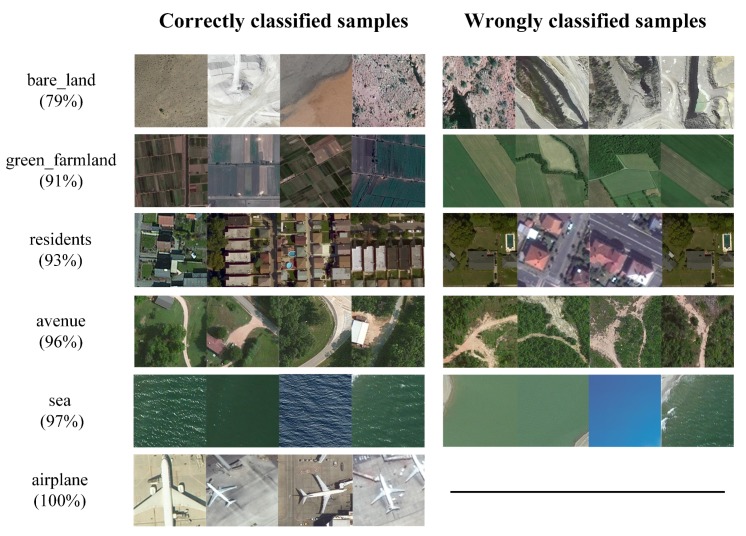
Theclassification results of VGGNet for RSI-CB (the numbers in brackets indicate the precision of classification).

Figure 14 reflects the test results based on the DCNN methods for the RSI-CB benchmark. Only a few categories had a precision less than 0.8, and the precision of nearly half of the categories was 0.9 or more. DCNN showed stronger recognition and non-linear fitting abilities than handcrafted methods. In addition, it benefitted from its database scale. Thus, DCNN can learn features from different categories well. The classification performance of each category was similar in different networks, but few categories showed unusual classification precision, which may be attributed to the random initialization and different structures of the models. VGGNet and ResNet achieved higher precision in each category, as compared to AlexNet and GoogLeNet, which further indicates that deeper networks are beneficial for improving model identification performance. Compared with other algorithms, the classification precision of VGGNet is the highest. Figure 15 shows the classification results of some categories based on VGGNet.

#### 4.5.3. Evaluation of Model Transfer Capability

According to the assumption that samples of the hypothesis space and real space conform to the same distribution, the representation of samples is the key point of data set quality. A general database evaluation index often evaluates a data set and considers the classification precision of the test set performance of models trained using a training set. The distributions of spatial features in real data and of test samples are different. Therefore, to improve model performance, we should allow the model to learn more data that are closer to the real distribution.

We selected 13 common categories in UC-Merced and RSI-CB as test sets (namely, UC-Merced-13 and RSI-CB-13), with each category containing 100 images according to the scale of UC-Merced, in order to verify the transfer ability of a model trained on our data set. We used the remaining images of RSI-CB for the 13 categories as the training set and selected AlexNet-Conv3 as the training model, considering the small number of samples. The training models were tested in UC-Merced-13 and RSI-CB-13.

Table 8 shows the results of the test on RSI-CB-13 and UC-Merced-13. The two data sets had differences in visualization, but were strongly consistent in the overall test results. As shown above, the model trained on our data set still had good transfer ability to UC-Merced, with a classification precision of 74.13%, which was approximately 12 percentage points less than that in RSI-CB, indicating that our data samples can represent real-world samples better. However, the result also provides evidence that, although the same categories were used and the same quantity of the same type of images between the two benchmarks were used, the accuracy of UC-Merced-13 was decreased. This result may indicate that, no matter how similar two benchmarks are, there always exists a feature gap between them. This means that, for the larger benchmark, it is not enough to learn all the features to train a learning model for the task of small benchmark classification. Furthermore, this explains the importance of scale, as a benchmark needs to fit the overall data characteristics as much as possible.

## 5. Conclusions and Future Works

Crowdsourced data have become a research focus, due to their remarkable features, such as real time classification, fast spread speed, robust information, low cost, and massive data. We proposed the RSI-CB benchmark, based on crowdsourced data, which provides new ideas and research directions for the establishment and improvement of remote sensing data sets. RSI-CB has six categories based on the Chinese land-use classification standard, where each category has several sub-categories. This is a significant improvement on the number of categories and images of other remote sensing data sets. We conducted a classification experiment on several traditional deep learning networks, showing that the classification accuracy of RSI-CB was higher than that of other data sets due to its higher spatial resolution.

RSI-CB can continue to expand, in terms of categories, quantity of objects, and diversity, based on the continuous emergence of more crowdsourced data, which are updated rapidly and are expected to increase massively in number. However, artificial work is required to obtain appropriate categories and images, given the complexity of the remote sensing image itself and the spatial resolution, which limit the construction speed, to some extent, resulting in a large amount of unavailable POI data.

At present, existing geographical data labels are manually marked, which requires a huge amount of human, material, and financial resources. In addition, the annotations come from different people, leading to the inconsistent quality and need for multiple checks, which increases the workload. When only using a small number of labeled samples, it is often difficult for a learning model to have a strong generalization ability when trained by them. On the other hand, when using only a small number of “expensive” tagged examples without using a large number of “cheap”, unlabeled examples, it is a great waste of data resources. In practice, unlabeled examples can be used to help improve learning performance, as long as a link between the unmarked sample distribution and the learning model can be reasonably established. Therefore, if we combine RSI-CB, which has been correctly marked with unlabeled or incomplete data to learn together, we can not only greatly increase the amount of data to increase the generalization ability of the model performance, but we can also correct errors in manual markers or achieve the correct marking of unmarked data, based on RSI-CB and weak supervised learning.

Experiments show that the RSI-CB benchmark uses different depth models to achieve good image recognition accuracy and has higher classification accuracy when transferring to other remote sensing image databases, which further proves that the RSI-CB classification benchmark plays an important role in image object recognition. Therefore, it is reasonable to believe that RSI-CB can be applied to different types of land recognition in remote sensing images, which can be of service in the relevant departments, making land use more reasonable.

Current remote sensing image databases, such as UCM and AID, have achieved good results in scene classification research. We believe that the scalable, high-quality, and diverse large-scale, multi-category RSI-CB can become a new and important remote sensing image benchmark for the development of new ideas, due to its application value and its method of construction.

We will extend this work by adding scene properties labels for relation reasoning or inferring on remote sensing images [63] combining with graph convolutional network [64].

## Figures and Tables

**Table 1:** Attributeinformation of POI data (collected from Open Street Map data; the left side of the table shows POI object attributes, and the right side is the corresponding sub-class under the attribute).

**amenity**	arts center, atm, bank, bar, bench, bicycle parking, bicycle rental, fountain...
**barrier**	bollard, gate, block...
**building**	apartments, building, hotel, house...
**emergency**	fire hydrant, defibrillator, ambulance_station, eemergency_ward_entrance...
**highway**	bus stop, crossing, motorway junction......
**historic**	memorial, monument...
**landuse**	commercial, industrial, farmland, forest, meadow...
**leisure**	park, picnic table, playground, swimming pool...
**man made**	antenna, flagpole, monitoring station, tower...
**natural**	peak, tree, grassland, tree...
**office**	accountant, company, government, adoption_agency...
**public transport**	platform, station, stop_area, stop_position...
**railway**	station, subway entrance, ventilation shaft...
**shop**	alcohol, antiques, art, books, clothes, convenience, hairdresser...
**sport**	gym, yoga...
**tourism**	artwork, gallery, hotel, museum...

**Table 2:** Theintersecting categories between the Chinese land classification standard and POI. The  vertical side of the table is the major class for OSM and the horizontal side is the land classification standard. Some OSM classes correspond to several categories in the land classification standard and vice versa.

	Land Classification	Residential	Commercial	Industrial, Manufacturing	Social, Institution, Infrastructure	Transportation	Leisure	Natural Resources Related	No Human Activity or Unclassifiable	Mass Assembly of People
OSM attributes										
amenity			√		√					
barrier										
building		√			√					
emergency		√								
highway						√				
historic										√
landuse		√						√		
leisure							√			
man_made				√						
natural								√		
office							√			
public_transport						√				
railway						√				
shop			√							
sport							√			
tourism							√	√		

Note: √ indicates that there is overlap between them.

**Table 3:** The sub-categories corresponding to the large categories in RSI-CB.

Large Class	Subclass
Agricultural land	green_farmland, dry_farm, bare_land
Woodland	artificial_grassland, sparse_forest, forest, mangrove, river_protection_forest, shrubwood, sapling
Transportation and facility	airport_runway, avenue, highway, marina, parking lot, crossroads, bridge, airplane
Water area and facility	coastline, dam, hirst, lakeshore, river, sea, stream
Construction land and facility	city_building, container, residents, storage_room, pipeline, town
Other land	desert, snow_mountain, mountain, sandbeach

**Table 4:** Different sub-classes and the number of images in each.

Categories	Number	Categories	Number	Categories	Number
airplane	351	dry_farm	1309	river	539
airport_runway	678	forest	1082	river_protection_forest	524
artificial_grassland	283	green_farmland	644	sandbeach	536
avenue	544	highway	223	sapling	879
bare_land	864	hirst	628	sea	1028
bridge	469	lakeshore	438	shrubwood	1331
city_building	1014	mangrove	1049	snow_mountain	1153
coast_line	459	marina	366	sparse_forest	1110
container	660	mountain	812	storage_room	1307
crossroads	553	parking lot	467	stream	688
dam	324	pipeline	198	town	355
desert	1092	residents	810		

**Table 5:** Comparison of RSI-CB with existing remote sensing data sets Nationla Land Cover Database (NLCD) database records pixels as categories, which is different from the recent database form). AID = aerial image data set; NWPU = Northwestern Polytechnic University

Database	Images	Categories	Average Per Category	Spatial Resolution (m)	Image Size (Pixels)
UC Merced	2100	21	100	0.3	256×256
SAT-4	500,000	6	83333	–	28×28
SAT-6	405,000	6	67500	–	28×28
NLCD	–	16	–	30	–
AID	10,000	30	333	0.5–8	600×600
NWPU RESISC45	31,500	45	700	0.2–30	256×256
RSI-CB	24,747	35	690	0.22–3	256×256

**Table 6:** Overall Accuarcy (OA) test on RSI-CB and UC-Merced.

Methods	Our Data Set (50%)	Our Data Set (80%)	UC-Merced (50%)	UC-Merced (80%)
SIFT	37.96±0.27%	40.12±0.34%	29.45±1.08%	32.10±1.95%
LBP	69.10±0.20%	71.98±0.36%	35.04±1.08%	36.29±1.90%
CH	84.08±0.26%	84.72±0.33%	42.79±1.06%	46.21±1.05%
Gist	61.74±0.35%	63.59±0.45%	45.38±0.70%	46.90±1.76%

**Table 7:** OA of training and test data sets using deep convolutional neural network (DCNN).

Methods	RSI CB	UC-Merced	SAT6
AlexNet-3ConV	–	78.69%/70.00%	98+%/97.53%
AlexNet	96+%/94.78%	74.82/65.53%	–
VGG-16	98+%/95.13%	–	–
GoogLeNet	98+%/94.07%	76.58%/67.62%	–
ResNet	98+%/95.02%	–	–

**Table 8:** Test results based on AlexNet-Conv3 for RSI-CB-13 and UC-Merced-13.

Data Set	Train	Test
RSI-CB-13	90%+	86.32%
UC-Merced-13	–	74.13%
